# ZnO/CuSe composite-mediated bandgap modulation for enhanced photocatalytic performance against methyl blue dye

**DOI:** 10.1038/s41598-023-46780-y

**Published:** 2023-11-09

**Authors:** Khalida Mubeen, Kashif Safeen, Afshan Irshad, Akif Safeen, Tayyaba Ghani, Wiqar H. Shah, Rajwali Khan, Khawaja Shafique Ahmad, Ryan Casin, Mohamed A. Rashwan, Hosam O. Elansary, Attaullah Shah

**Affiliations:** 1 Department of Physics and Applied Mathematics, Pakistan Institute of Engineering and Applied Sciences (PIEAS), Nilore, Islamabad, 45650 Pakistan; 2 Department of Physics, Abdul Wali Khan University, Mardan, 23200 Pakistan; 3 Center for Mathematical Sciences, PIEAS, Nilore, Islamabad, 45650 Pakistan; 4 National Institute of Lasers and Optoelectronics College, Pakistan Institute of Engineering and Applied Sciences, Nilore, Islamabad, 45650 Pakistan; 5 Department of Physics, University of Poonch Rawalakot, Rawalakot, AJK 12350 Pakistan; 6 Department of Metallurgy and Material Engineering, Pakistan Institute of Engineering and Applied Sciences (PIEAS), Nilore, Islamabad, 45650 Pakistan; 7 Department of Physics, Faculty of Basic and Applied Sciences, International Islamic University, H-10, Islamabad, Pakistan; 8 Department of Physics, University of Lakki Marwat, Lakki Marwat, KP 28420 Pakistan; 9 Department of Botany, University of Poonch Rawalakot, Rawalakot, AJK 12350 Pakistan; 10 School of Public Health, University of California, Berkeley, 2121 Berkeley Way, Berkeley, CA 94704 USA; 11 Department of Agricultural Engineering, College of Food and Agriculture Sciences, King Saud University, 11451 Riyadh, Saudi Arabia; 12 Plant Production Department, College of Food and Agriculture Sciences, King Saud University, 11451 Riyadh, Saudi Arabia

**Keywords:** Materials science, Physics

## Abstract

The removal of toxic dye pigments from the environment is of utmost importance since even trace amounts of these pollutants can lead to harmful impacts on ecosystems. Heterogeneous photocatalysis is a potential technique for eliminating microbiological, inorganic, and organic pollutants from wastewater. Here, we report the band gap alteration of ZnO by making its composites with CuSe to enhance photocatalytic activity. The purpose is to develop metal oxide nanocomposites (ZnO/CuSe) as an effective and efficient material for the photodegradation of methyl blue. The photocatalysts, ZnO nanorods, CuSe, and ZnO/CuSe nanocomposites of different weight ratios were synthesized by the simple and cost-effective technique of precipitation. UV–Vis spectra verified that the ZnO/CuSe photocatalyst improved absorption in the visible region. The optical bandgap of ZnO/CuSe nanocomposites reduced from 3.37 to 2.68 eV when CuSe concentration increased from 10 to 50%. ZnO/CuSe composites demonstrated better photocatalytic activity than ZnO when exposed to UV–visible light. The pure ZnO nanorods could absorb UV light and the nanocomposites could absorb visible light only; this was attributed to the transfer of excited high-energy electrons from ZnO to CuSe.

## Introduction

The development of energy and environmental technologies is crucial for achieving sustainable growth. However, toxic industrial waste is a persistent source of environmental problems due to its hazardous, persistent, and bio-accumulative nature. Therefore, effective solutions must be developed to tackle these issues^1–3^. The increasing concerns over water contamination make it even more urgent to find efficient and low-energy-intensive water treatment methods^4^. To treat water with photocatalysis, many pollutants have been observed^5^. Along with actual wastewater, frequent target contaminants include dyes like acid red 114, ethyl violet, and methyl blue (MB) as well as aromatic chemicals like phenol, toluene, and chlorobenzene^6^. The investigation of the degradation of dyes is really attractive because traditional technologies typically have a hard time breaking them down^7^. As a sustainable technology photocatalysis has attracted full attention due to its capability to solve water-related environmental problems^8,9^. Because of its cheap cost and innocuous nature, the heterogeneous photocatalytic process has attracted a lot of interest among numerous wastewater treatment technologies, notably for the removal of organic pollutants. Several studies have currently shown that direct solar-based photocatalytic degradation of organic pollutants using semiconductor photocatalysts is one of the most efficient ways to combat future pollution^10,11^. Many semiconductor photocatalysts have been invented and developed to alleviate environmental pollution such as TiO_2_, ZnO, WO_3_, and CuSe^12,13^. ZnO is regarded as one of the most favorable semiconductor photocatalysts because of its excellent catalytic activity, high chemical stability, and biological compatibility^8,14^. The wide bandgap of ZnO, however, restricts its absorption of visible light, which makes up just 5% of solar radiation, and hence restricts its photocatalytic activity. Hence, there is still a challenge in efficiently utilizing solar energy in photocatalytic applications. To increase the photocatalytic performance of ZnO, a variety of techniques have been used, including the creation of acceptable sizes and morphologies, noble metal loading, atom doping, and the formation of heterostructure composites, among others^15–17^. The addition of transition metal ions like iron, nickel, and cobalt to ZnO has been shown to increase its photocatalytic activity by intr2oducing new energy levels within the bandgap, reducing its bandgap width, and promoting the separation of photogenerated electron–hole pairs^18,19^. Similarly, doping with rare earth metal ions like lanthanum, cerium, and gadolinium has also been proven to enhance ZnO’s photocatalytic activity. This is attributed to the unique electronic structures of these ions and their strong absorption in the visible light region^20^. Despite the potential benefits of doping with transition in metal oxide, there are often accompanying issues that limit the photocatalytic performance. For instance, the introduction of dopants can create secondary oxide phases and unwanted defects in the crystal structure, which can reduce the photocatalytic activity and stability of the resulting composite material^21^. Thus, there is a need to investigate other systems. Recently, p–n heterojunction photocatalysts have gained much attention because of the effective separation of photo-initiated holes and electrons. At the interface of two semiconductors, p-type and n-type semiconductor coupling results in an inner electric field. The design and development of p-n heterostructure composites based on the coupling of chalcogenide materials and ZnO, in particular, is an effective option for improving its photocatalytic response under UV/Vis irradiation^22^. Furthermore, in bare ZnO, the recombination of electrons and holes occurs quickly. These are the considerable hindrances of the ZnO photocatalyst in practical use. To solve these drawbacks, heterostructures are formed with other semiconductors to improve electrons and hole mobility, reduce recombination, and increase the light absorption zone^15^.

The choice of ZnO/CuSe as a heterojunction is driven by its advantageous band alignment, which promotes efficient charge separation, a key factor in enhancing photocatalytic activity^23^. Additionally, CuSe exhibits notable photocatalytic properties, particularly under visible light, making it well-suited for environmental applications where visible light is abundant. Its relative photostability and compatibility within the nanocomposite system further contribute to its selection. Practical factors, including cost-effectiveness and material availability, also make CuSe a compelling choice for researchers aiming to develop effective and sustainable photodegradation processes^24^.

CuSe is a p-type semiconductor with a narrow bandgap that can be combined with the n-type wide bandgap semiconductor ZnO to enhance photocatalytic efficiency. Due to its complicated structure and valence state, copper selenide (CuSe), a significant semiconductor, has been extensively used in superconductors, solar cells, optical filters, thermoelectric and photoelectric transformers, and solar energy systems^25^ M. Iqbal et al.^26^ demonstrated the remarkable photocatalytic performance of CuSe/GO heterojunction in degrading organic pollutants under visible light. Additionally, Cheng et al.^27^ highlighted the potential of CuSe/MoSe_2_ heterojunction for water purification. These findings underscore the growing interest in harnessing metal chalcogenides like CuSe for environmental applications^28^. The resulting nanocomposite photocatalyst consists of p-type CuSe and n-type ZnO, which can form an interface to enhance photocatalytic activity when exposed to visible light. Essentially, the wide bandgap of ZnO and the narrow bandgap of CuSe work as co-catalysts.

In this study, the precipitation technique was used to synthesize ZnO/CuSe nanocomposites. These nanocomposites are used to study the degradation of MB. The X-ray diffraction (XRD) was employed to determine the structure properties. Scanning electron microscopy (SEM) and energy-dispersive X-ray spectroscopy (EDX) investigations were employed to determine the surface morphology and chemical characteristics of the nanocomposites, respectively. The photodegradation activity of samples was examined through the degradation of MB dye.

## Material preparation

All chemicals were commercial and analytically pure without further purification. Zinc acetate dihydrate (Zn (CH_3_COO)_2_·2H_2_O, 99.9%), and copper chloride (CuCl_2_, 99.9%) were purchased from Sigma-Aldrich. Sodium hydroxide (NaOH, 99%) was used as a precipitating agent, and selenium powder (Se) and MB dye used in photodegradation investigation were purchased from Sigma-Aldrich. Ethanol (Merck), and distilled water is used for the preparation of the solution.

In a typical procedure, we took 0.5 M zinc acetate dihydrate solution and stirred the solution for about 15 min. NaOH of about 01 M solutions was added dropwise and heated at 80 °C overnight. Water and ethanol were used to wash the produced ZnO. The nanorods were dried at 80 °C and calcinated at 450 °C for 1h. Finally, pure ZnO nanostructure was collected. Quantitatively, made the solution of 12 M sodium hydroxide (NaOH) and 2.5 g of selenium powder (Se) in deionized water at 40 °C. The addition of NaOH helps to increase the solubility of Se and the production of CuSe. 0.2 M of copper chloride was dissolved in 50 ml of deionized water. The reduction potential of hydroxyl ion and elemental selenium is about + 0.82 eV and − 0.67 eV. Initially, elemental selenium (Se) was transformed into Se^2−^, leading to a substantial monomer concentration. In the first stage, hydroxyl ions react with copper ions (Cu^2+^), forming a transparent soluble complex solution^29^. This complex formation effectively reduces the concentration of Cu^2+^ ions and prevents the precipitation of CuSeO_3_. The overall chemical reaction occurring during the entire synthesis of nanoplates can be expressed as follows

1 $$2{\text{Cu}}^{{2 + }} + 4{\text{OH}}^{ - } \to 2{\text{CuO}} + 2{\text{H}}_{2} {\text{O}}$$

2 $$4{\text{CuO}} + 4{\text{Se}}^{2 - } \to 4{\text{CuSe}} + 8{\text{OH}}^{ - }$$

3 $$2{\text{Cu}}^{2 + } + 6{\text{OH}}^{ - } \to 2{\text{Cu}}\left( {{\text{OH}}} \right)_{3}^{ - }$$

4 $${\text{Cu}}\left( {{\text{OH}}} \right)_{3}^{ - } + {\text{Se}}^{2 - } \to {\text{CuSe}} + {\text{OH}}^{ - }$$

Black precipitators were formed when solutions were mixed and stirred for 2 h at room temperature. The obtained sample was separated by centrifuge, washed with deionized water and ethanol three times, and dried at 80 °C.

### Preparation of the ZnO/CuSe nanocomposites

**Figure 1:**
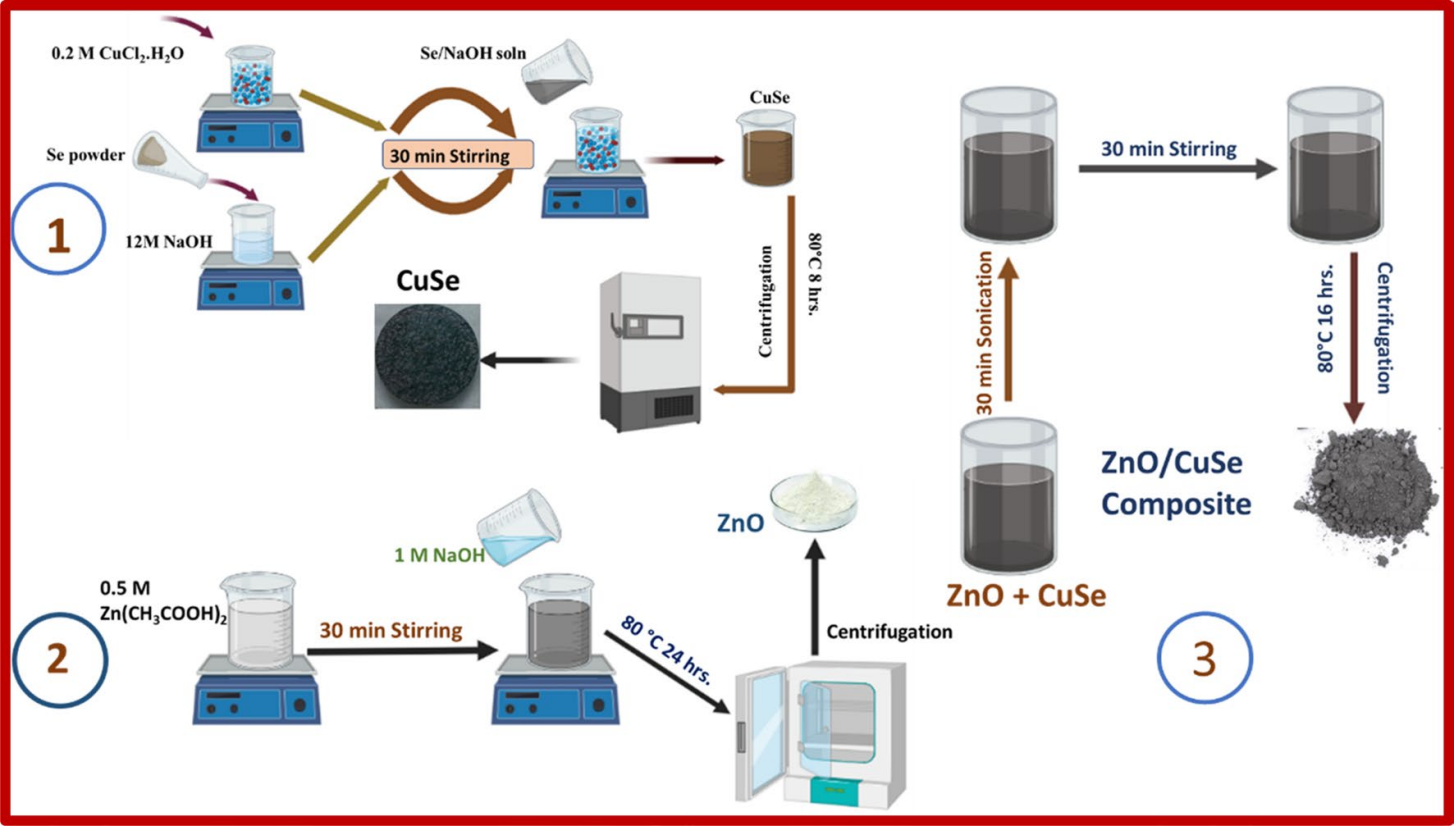
The schematic representation of ZnO/CuSe heterojunction photocatalyst.

The following procedure is used to synthesize ZnO/CuSe nanocomposites with varying CuSe/ZnO weight percentages of 10, 30, and 50%. To ensure uniform distribution and thorough mixing of the components, the resulting mixture underwent both sonication and stirring, lasting 30 min each, at room temperature. Finally, to eliminate the methanol solvent, the mixture of nanocomposites was exposed to an oven set at 60°C for a duration of 16 h. The specific characteristics and properties of the resulting ZnO/CuSe nanocomposites are contingent on the precise ratios and types of precursor materials employed, as well as the intricate details of the synthesis conditions. The flow chart of the synthesis process of ZnO/CuSe heterojunction is shown in Fig. 1. The synthesized samples are labeled as pure ZnO, ZnO-10% CuSe (S-1), ZnO-30% CuSe (S-2), ZnO-50% CuSe (S-3) and pure CuSe.

### Characterizations

The morphology of the composites was observed by scanning electron microscope (FESEM, MAIA-3, TESCAN), and an energy dispersive spectrometer (EDX) was used to analyze the composition of the sample. X-ray powder diffraction using CuKα radiation (XRD) and Raman spectrometry were used to investigate the crystal phases and vibration modes of the samples, respectively. UV–visible spectrometer (UV–Vis) was used to study the optical properties of samples. To test the sample's photocatalytic efficiency, the absorbance of the MB solution was measured by a UV–Vis spectrophotometer at the wavelength of maximum absorption.

### Preparation of photocatalytic degradation samples

For the degradation experiments, a 400 W Xenon lamp was used for UV light irradiation. The light produced by the xenon lamps includes broad-spectrum wavelengths from 100 to 1100 nm: UV light (100–400 nm), visible light (400–700 nm), and near-infrared light (700–1100 nm). However, here we used the xenon lamp as a visible light source. We study the photocatalytic activity under the visible light. The sample was kept 15 cm away from the light source to avoid heating with intense irradiations. To investigate the photocatalytic activity of the composites, MB was used as a photocatalytic degradation. The reaction temperature was taken to room temperature. To make the suspension of 0.1 mg photocatalyst and 25 mL MB (12 mg/L or 12 ppm) adsorption/desorption equilibrium was established by stirring in the dark for 20 min. The absorbance of MB was measured at given time intervals under the UV–Vis light (400 W) With UV–Vis absorption spectra at maximum absorption wavelengths of 664 nm. Using the following equation finally, the photocatalysis degradation (ƞ) was measured by the photocatalytic activity of the investigated system.

5 $$\eta = \left( {1 - \frac{C}{{C_{0} }}} \right) \times 100$$

where C_o_ and C are the initial and final MB absorbance, respectively

## Results and discussion

**Figure 2:**
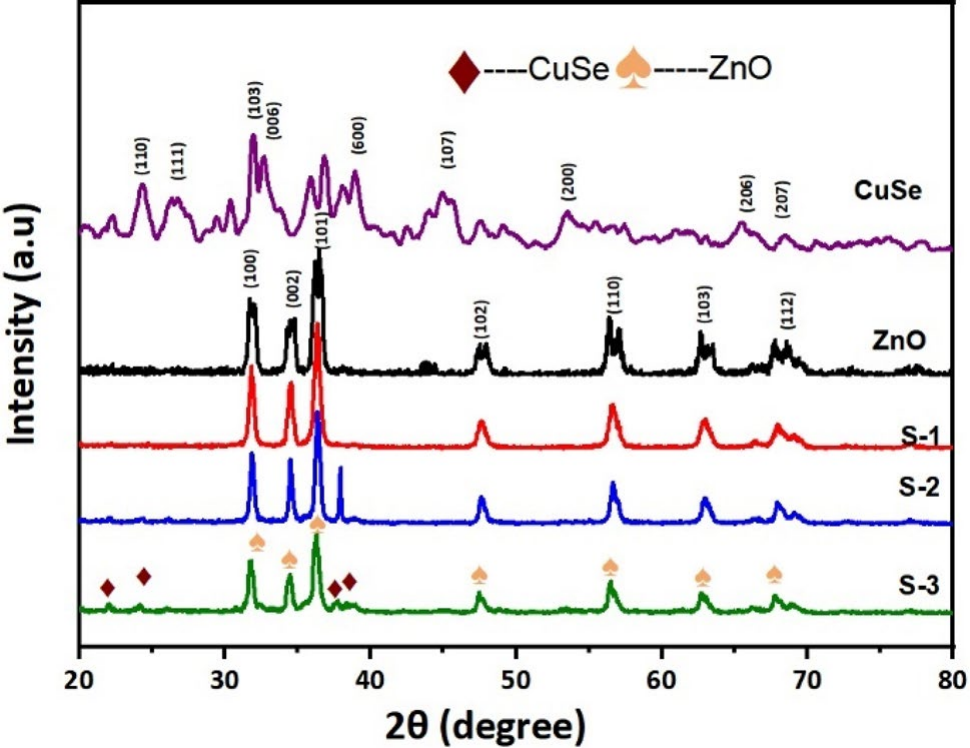
XRD pattern of CuSe, ZnO-10% CuSe (S-1), ZnO-30% CuSe (S-2), ZnO-50% CuSe (S-3) and ZnO.

For phase identification of a crystalline material, XRD is a quick analytical technique used and the quality of the crystal can be determined by the intensity of XRD peaks which is shown in Fig. 2. The XRD of the pure ZnO displays intense diffraction peaks, which indicate the wurtzite phase structure, according to JCPDS card (01-75-1526)^30,31^. Hexagonal copper selenide has a good fit with the position and intensities of diffraction peaks. The lattice parameters of tetragonal CuSe are a = b = 3.9390 Å and c = 17.25 Å. It is found that the XRD pattern of CuSe is well-matched with the JCPDS data (00-034-0171). These strong peaks demonstrate the great crystallinity and purity of the produced samples. S-2 and S-3 show illustrated XRD patterns of the ZnO/CuSe composite. The diffraction peaks appeared at 2θ values of 25.2°, 27.8°, 28.1°, 31.1°, 40.1°, and 42.8°, which are well indexed to the (101), (200), (111), (210), (220), and (002) planes, respectively, are the characteristics peaks of CuSe in the nanocomposite. Due to the lower weight percentage of CuSe, Sample S-1 shows the only correspondence peaks of ZnO. CuSe has crystallinity even in composites, according to the XRD pattern, which contains a CuSe peak^32–34^.

Utilization of the Scherrer formula^35–37^ indicated that the average crystallite size of CuSe is ∼ 26.9 nm and the crystallite size of ZnO/CuSe nanocomposite varied from 26.5 to 32.5 nm. The size of the nanocomposite has increased in comparison to the pure CuSe nanostructure (Table 1). The size of the nanocomposites increased due to the aggregation and agglomeration of nanoparticles within the nanocomposites. Another factor that also affects the size of the nanocomposites is the degree of dispersion of the nanoparticles. If the materials are not well dispersed then the size of nanocomposites increases.

**Table 1:** Grain size calculation of the synthesized samples.

Samples	2 θ (degree)	FWHM (rad)	Grain size (nm)
Pure ZnO	36.46	0.3263	25
Pure CuSe	32.59	0.3660	26.9
S-1	36.33	0.3355	26
S-2	36.35	0.3050	28.6
S-3	36.16	0.2440	32.5

**Figure 3:**
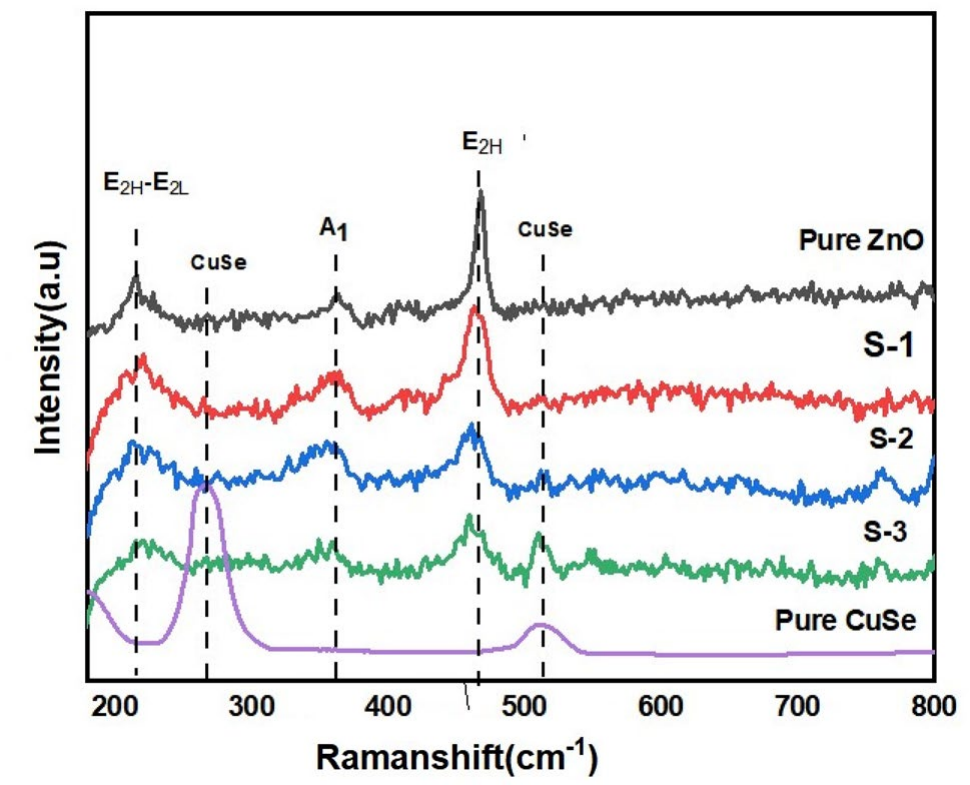
Raman spectrum of CuSe, ZnO-10% CuSe (S-1), ZnO-30% CuSe (S-2), ZnO-50% CuSe (S-3) and ZnO.

Additionally, Raman measurements were carried out to analyze the structural data supporting the XRD investigation, and the results are presented in Fig. 3. Raman active phonon modes at 333.5 cm^−1^ and 184.7 cm^−1^, corresponding to the E_2_ (high) and A_1g_ modes, were found for ZnO nanorods. These peaks match the reported wurtzite-like structure of ZnO in the literature. Similarly, in ZnO/CuSe spectra, besides ZnO peaks, two more peaks can be seen at 495 and 256 cm^−1^ related to CuSe. With increasing the percentage of CuSe in nanocomposites, the peak Intensity (Eg) at 185 cm^−1^ and 438 cm^−1^ are gradually decreased. Peaks related to any impurity have not been observed in Raman as well as XRD analysis which indicates the high purity of our synthesized samples^8,38^.

**Figure 4:**
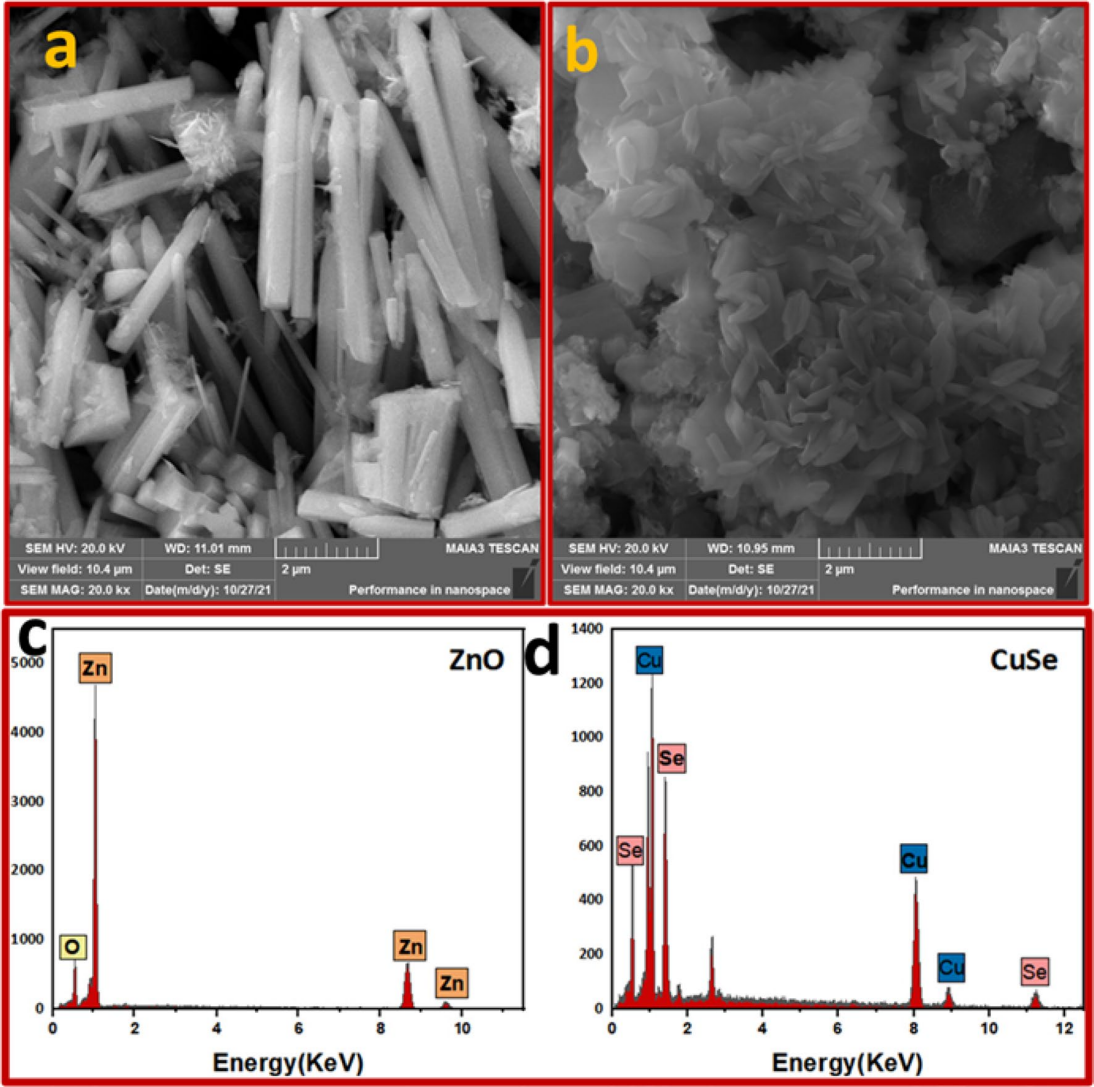
SEM images of (**a**) ZnO (**b**) CuSe, EDX spectra of (**c**) ZnO, and (**d**) CuSe.

The morphology development and nanostructures of ZnO, CuSe, and ZnO/CuSe composites were studied by SEM, as shown in Fig. 4. ZnO nanostructure, grown at 80 °C by chemical method, is a powder made up of both thick and thin rods, as depicted in Fig. 4a. The typical diameters of thick and thin rods are 500 nm and 120 nm, respectively have clear conical hexagonal edges and smooth surfaces. Figure 4b revealed the irregular particles-like morphology of pure CuSe nanostructure with an average width of 50 nm. The particles form big aggregates with some merged surfaces and little particles on the surfaces. A greater surface area is seen on the rough edges of NPs due to their roughened surfaces. The agglomeration of the particles may be brought on by the forces of Wander Walls, which cause the particles to unite, according to Shah et al.^34^. The EDX spectrum of ZnO contains Zn and O Fig. 4c. Similarly, for the CuSe NPs, the NP purity is high and there is no impurity such as oxygen Fig. 4d. The elemental analysis results, which concurred with the XRD findings, represented that the atomic ratio of Cu to Se was nearly 1:1.

**Figure 5:**
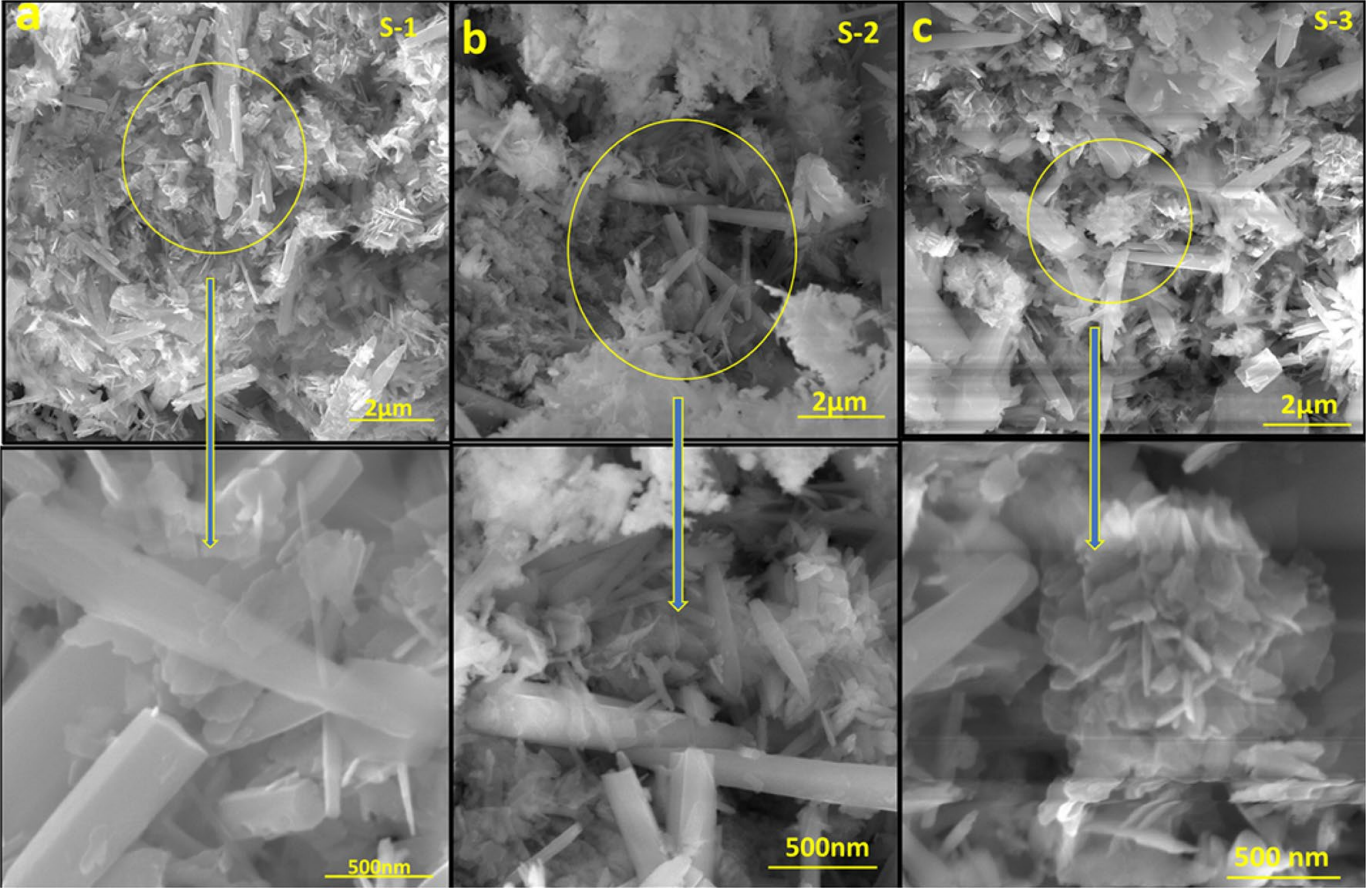
SEM images of (**a**) ZnO-10% CuSe (S-1), (**b**) ZnO-30% CuSe (S-2) and (**c**) ZnO-50% CuSe (S-3).

Figure 5a–c represents the effect of CuSe nanoparticles in addition to the morphology of ZnO. The morphology of individual components was shown to alter during composite creation (ZnO and CuSe). Several clusters are formed when the CuSe particles that are gathered around the ZnO nanorods agglomerate. ZnO has a rod-like shape and a range of sizes, with an average length of 3–5 mm and a diameter of 20–50 nm. CuSe shows mixed morphology that contains nanoparticles and distinctive arrangements that form sufficient active sites which is beneficial for photocatalysis. SEM images show the impact of CuSe concentration on the structure and morphology of the composite nanostructures. CuS is evenly dispersed on the ZnO nanorods’ surface without altering its shape, demonstrating successful structural integration during the production process as seen in Fig. 5a with 10%CuSe. The different-sized nanorods and particles are tightly agglomerated, with smaller spaces and cavities between the grains, as can be seen in the SEM pictures for 30 and 50% CuSe. The CuSe nanoparticles pack into the gaps within the ZnO nanostructure, causing the size to decrease. These findings show that the addition of CuSe causes ZnO nanorods to fragment, changing the material's dimensional characteristics. The texture of the nanocomposites has a pronounced impact on their photocatalytic activity. Nanocomposites with finer textures and smaller particle sizes generally exhibit significantly larger surface areas. This expanded surface area provides a multitude of active sites where photocatalytic reactions can occur, resulting in enhanced photocatalytic activity.

CuSe, in addition to ZnO, caused changes in its shape, demonstrating how much the concentration of Cu^2+^ ions affect ZnO crystal growth rate nanomaterials. ZnO nanorods turn into small pieces with aggregated CuSe nanoparticles forming a cluster. As a reference, the SEM images of pure CuSe, nanoparticles, ZnO nanorods, and composites along with their elemental mapping are shown as supplementary information in Fig. S1.

**Figure 6:**
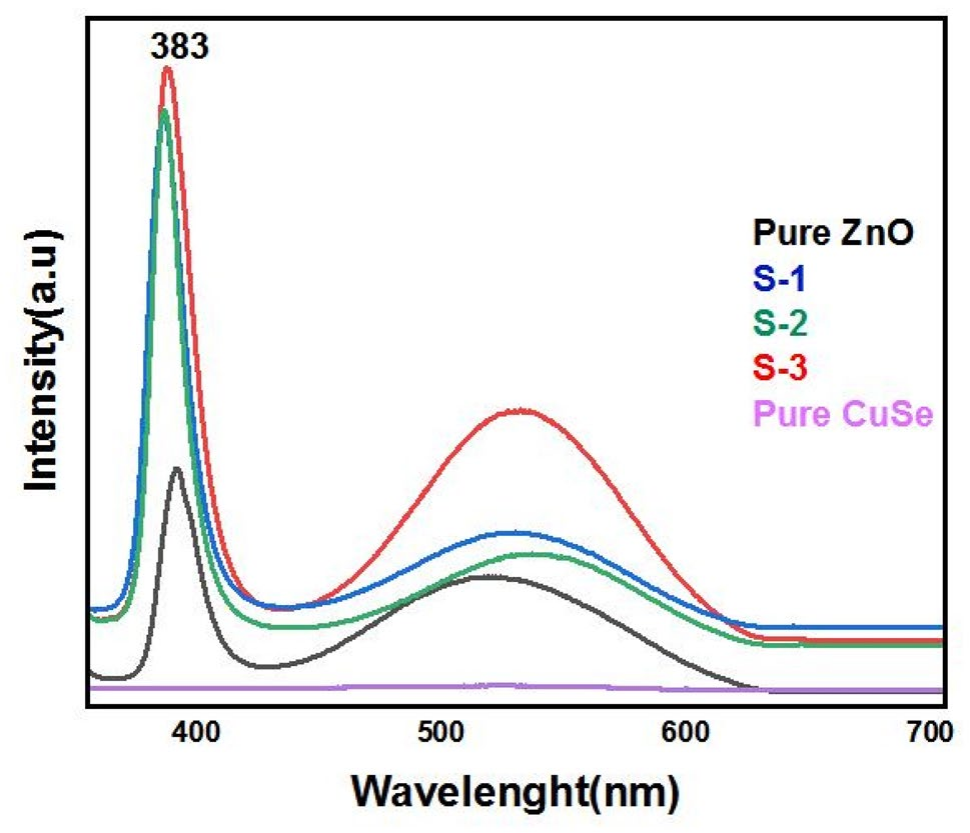
PL spectrum of pure ZnO, pure CuSe, and ZnO/CuSe nanocomposites.

To examine the optical characteristics of ZnO nanocomposites, a photoluminescence (PL) investigation was conducted at room temperature. Figure 6 displays the PL spectra. Pure ZnO nanorods' PL spectra showed two peaks. In the near-band edge of ZnO, free excitons recombined to produce the peak of the intense emission fixed at 383 nm in the UV range. It was widely believed that oxygen vacancies were responsible for the other peak, which showed a broad emission near the green band^39^. Electron–hole pair recombination and photoluminescence are closely related processes. Photo-generated electron–hole pairs recombine more slowly the weaker the PL intensity is. The fact that CuSe nanoflakes have better optical characteristics than pure ZnO nanowires is shown by the fact that their PL intensities are lower. Additionally, multiple electron traps are provided using ZnO/CuSe nanocomposites to prevent the recombination of electron–hole pairs. Within ZnO/CuSe nanocomposites, several electron traps are present, each associated with three distinct structures. These structures include ZnO nanoparticles characterized by surface defects, CuSe nanoparticles with inherent trap states, and the interfaces or heterojunctions formed between them. These traps represent localized energy states within the bandgap, serving to capture and temporarily retain electrons. Notably, these traps possess varying energy levels and kinetics, which effectively hinder swift electron–hole recombination. As a result, they play a pivotal role in enhancing photocatalytic efficiency by optimizing the utilization of charge carriers for catalytic reactions^40,41^. ZnO/CuSe nanocomposites possessed higher optical characteristics and a reduced recombination rate, which was evident from comparing their PL intensities.

**Figure 7:**
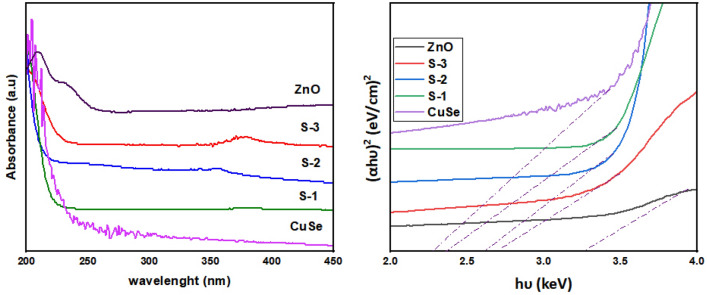
(**a**) UV spectrum and (**b**) Tauc plot of nanocomposites.

All samples were exposed to UV–Vis spectroscopy experiments to examine the optical characteristics of CuSe and ZnO-based nanocomposites shown in Fig. 7a. Due to high bandgap energy, ZnO shows maximum absorption in the UV region^42,43^. However, with the addition of CuSe, the absorption capability of ZnO gradually moved toward the visible region, which shows a decrease in the bandgap of ZnO/CuSe nanocomposites as compared to pure materials^44^.

A crucial variable in photocatalysis is the band gap of the materials. Different band gaps are evident from the variance in the absorbance of the various catalysts. The Tauc plots are used to determine the corresponding bandgap energies of the nanocomposites which are as follows^14,45,46^.

6 $$\alpha h = A\left( {h - E_{g} } \right)^{n}$$

where A and n are constants, *hv* and *E*_*g*_ are the incident photon energy and the bandgap energy, respectively, and α is the absorption coefficient^47,48^.

The weight percent ratios of CuSe are displayed as a function and used to calculate the bandgap values of the nanocomposites. Figure 7b shows a decrease in the bandgap energy of ZnO by CuSe in composites. These values are approximate to be 3.37, 2.62, 2.57, 2.42, and 2.38 eV, respectively, for pure ZnO, S-3, S-2, S-1, and pure CuSe. In this phenomenon, the superficial bandgap of the semiconductor decreases as the absorption edge gets shifted to a longer wavelength. The red shift in bandgap energies shows that adding CuSe to pure ZnO can change its optical characteristics, making it ideal for photocatalytic applications^49^.

### Photocatalytic activity

The degradation activity of MB was tested using an Xeon lamp. The UV–vis absorbance of MB showed a strong peak at 664 nm. Firstly, UV–visible light sources offer precise control over the wavelength and intensity of light, enabling systematic and reproducible experiments. While visible light is the primary cause of MB degradation, UV light may also play a role, and researchers need to differentiate their effects. Secondly, UV–visible spectroscopy allows for a deeper understanding of the degradation mechanisms. By studying the absorption and spectral changes of MB in the presence of nanocomposites under UV–visible light, researchers can gain insights into the underlying chemical reactions and kinetics involved. Without a photocatalyst, the photocatalytic activities of the ZnO/CuSe heterostructures are analyzed through the photodegradation of MB. The pH in all catalyst solutions remains stable or constant throughout the degradation process. With time, the absorbance of the MB aqueous solution decreased after being exposed to UV–visible light, showing the MB had photodegraded. As a reference, the data from the degradation of MB solution without catalysis under UV–Vis irradiation are shown as supplementary information in Fig. S2.

**Figure 8:**
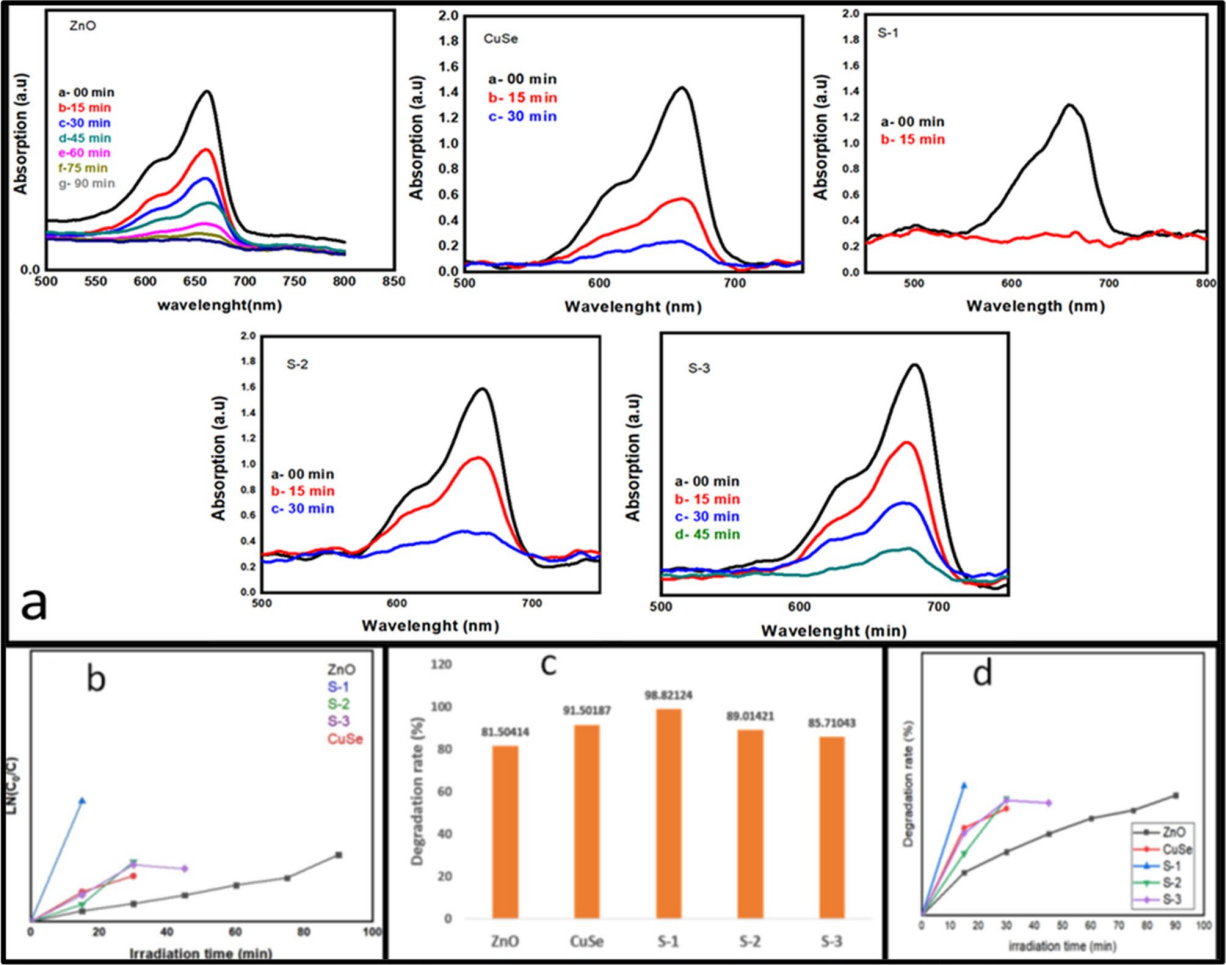
(**a**) Degradation of MB with ZnO/CuSe heterostructures as a photocatalyst, (**b**) degradation rate for ZnO/CuSe heterostructures, (**c**) photodegradation efficiency, (**d**) comparison of catalytic activity of photocatalysts.

Figure 8a depicts the photodegradation activity of the prepared samples tested against MB. When ultraviolet or visible light is absorbed, on semiconductor surfaces, it generates valance band holes and conduction band electrons that act as catalysts in oxidation and reduction reactions. After carrying out the reaction under irradiation, a significant drop in the absorbance of dye MB was noticed. Furthermore, there was negligible photocatalytic degradation of MB by ZnO, showing that ZnO has a limited capacity to remove MB from water. Under visible light, all of the CuSe-based samples demonstrated effective MB removal. Under irradiation, the ZnO/CuSe nanocomposites showed a substantial increase in photocatalytic efficiency as compared to dark conditions. Under the UV–visible irradiation, the degradation of binary ZnO/CuSe nanocomposites is much more quickly than that of pure samples^55^. Results for various photocatalysts' photocatalytic efficiency are shown in Fig. 8b. Compared to pure ZnO, all ZnO/CuSe composite samples exhibit improved photodegradation abilities. The enhanced photocatalytic ability was associated with the vigorous formation of p–n heterojunction consisting of p-type CuSe and n-type ZnO. The prolongation in UV absorption and the recombination rate of photogenerated electron–hole pair, manifest the potential of p–n heterostructures to be employed for photocatalytic processes^56^. Figure 8a–d illustrates the outcomes of the degradation process. The data reveals a notable escalation in the degradation rate of the dye as the concentration of CuSe in ZnO increases. The degradation of the dye when using pure ZnO takes nearly six times longer compared to when employing the nanocomposite. This indicates a significantly enhanced photocatalytic efficiency in the nanocomposite compared to both pure ZnO and CuSe materials. Photodegradation efficiencies of ZnO, CuSe, and ZnO/CuSe nanocomposites for MB, under irradiation, were 81.5%, 91.5%, 98.8%, 89.0%, and 85.7%, respectively, as shown in Fig. 8c. Table 2 compares the photocatalytic activity for MB at various photocatalysts with existence work.

**Table 2:** Comparison table for degradation of dye.

Catalyst	Model dye pollutant	Degradation time (min)	Degradation rate (%)	Source of light	References
GO/CuSe	Methylene green (MG)	45	89	Sunlight	^26^
ZnO-g-C_3_N_4_@PET	Methylene blue (MB)	120	92	Visible light irradiation	^50^
CuSe nanoflakes	methylene blue (MB)	90	76	Sunlight	^13^
ZnO/Bi_2_WO_6_	methylene blue (MB)	120	97	250 W Hg lamp	^51^
ZnO–CdS	RhB dye	80	98	solar light	^52^
ZnO/SiO_2_	methylene blue	120	95	UV-B lamp	^53^
ZnO/CuO	Methylene Blue	120	97	Sunlight	^54^
ZnO/CuSe	Methyl blue	15	98.8	Xe lamp	Present work

Figure 8d shows the photodegradation rate versus irradiation time (t) for the photocatalytic reaction. With increases in the concentration of CuSe, the photocatalytic activity decreases, and S-1 is the best for photocatalytic activity.

Various scavengers were introduced to the photocatalytic process, namely EDTA, benzoquinone, methanol, and isopropyl alcohol. The outcomes of the photocatalytic degradation of MB, in the presence of different scavengers, are shown as supplementary information in Fig. S3. The degradation of MB by different catalysts and their reusability and stability is given in supplementary information as Figs. S4 and S5.

### Kinetics of the photodegradation

Since n-type ZnO and p-type CuSe have very different Fermi energy levels, electrons, and holes will move between the two materials until the two Fermi levels are at the same level. Therefore, a new p-n heterojunction will be created when p-type CuSe and n-type ZnO are connected. The findings show that CuSe and ZnO may both be stimulated by visible light and produce photogenerated electron–hole pairs. The p-n heterojunction allows the photogenerated electrons from CB of CuSe to move to CB of ZnO while the photogenerated holes from ZnO move to VB of CuSe. The ZnO and CuSe photogenerated electrons and holes will successfully be separated in this manner. The effective separation of photogenerated carriers requires the construction of a heterojunction. The separation of photogenerated charge carriers can be facilitated by the p-n heterojunction system created between CuSe and ZnO. When exposed to UV radiation, CuSe and ZnO exhibit photoexcitation leaving behind holes in the valance band (VB). Since ZnO's conduction band (CB) and VB are lower than those of CuSe, ZnO's VB photoexcited holes transfer to the VB of CuSe, while the CB photoexcited electrons transfer to the CB of ZnO. The higher photocatalytic activity of the ZnO/CuSe heterostructures is due to the role of p-type CuSe as the electron reservoir for the n-type ZnO, such as;

7 $${\text{CuSe}} + hv \to {\text{CuSe}}\left( {h^{ + } + e^{ - } } \right)$$

8 $${\text{CuSe}}\left( {h^{ + } + e^{ - } } \right) + {\text{ZnO}} \to {\text{CuSe}}\left( {h^{ + } } \right) + {\text{ZnO}}\left( {e^{ - } } \right)$$

Superoxide radicals ($.{\text{O}}_{2}^{ - }$)will be created when the oxygen molecules in the solution are coupled with the electrons. In a consequent reaction, the ${\text{O}}_{2}^{ - }$ can create hydroxyl radicals (OH), a potent oxidant that can damage the dye molecules. CuSe holes can immediately oxidize and degrade MB. The separation of the photoexcited carriers as a result of the charge transfer improves the efficiency of the photocatalytic reaction.

**Figure 9:**
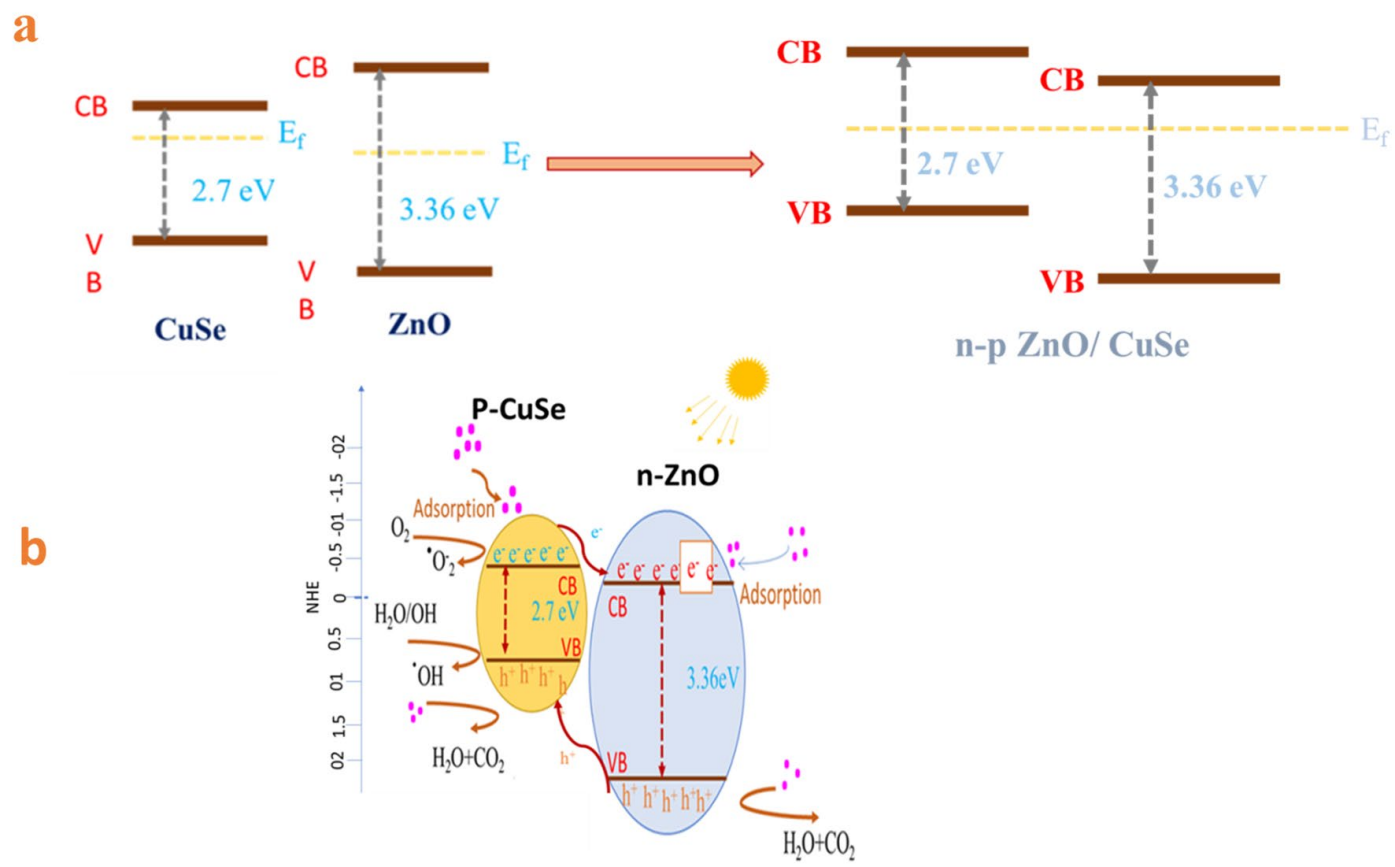
Schematic diagram of (**a**) the bandgap energy structure of the ZnO/CuSe heterojunction and (**b**) the mechanism of degradation of MB by ZnO/CuSe photocatalyst.

According to the aforementioned findings, an apparent photodegradation process for the ZnO/CuSe nanocomposites is schematically presented in Fig. 9a,b. When compared to ZnO nanorods, ZnO/CuSe nanocomposites perform more photocatalytic degradation, and this improvement is due to two factors. One, ZnO/CuSe nanocomposites can absorb visible light. Consequently, more electrons and holes will be produced. Secondly, the separation of photogenerated carriers is facilitated by the heterojunction created by CuSe and ZnO^8^. The valence band (VB) and the conduction band (CB) edge position of ZnO and CuSe were estimated according to their absolute electronegativity and the band edge positions can be determined by the following Eq. (9):

9 $${\text{E}}_{{{\text{CB}}}} = {\upchi } - {\text{E}}_{{\text{e}}} - 0.5{\text{E}}_{{\text{g}}}$$

where E_CB_ is the conduction band edge potential, χ is the electronegativity of the semiconductor, expressed as the geometric mean of the absolute electronegativity of the constituent atoms. E^e^ is the energy of free electrons on the hydrogen scale ca. 4.5 eV. The approximated CB potentials of CuSe and ZnO are − 0.7 and − 0.2 eV versus NHE and their band gaps are 2.38 and 3.36 eV. The kinetics of the photodegradation Langmuir–Hinshelwood model used to analyze the ZnO/CuSe photocatalysts via the pseudo-first-order term Eq. (10).

10 $$Ln\left( {\frac{{C_{0} }}{C}} \right) = kt$$

where C_0_ and C represent the initial and final concentrations of MB at the initial and final time, and k (min^−1^) is the photodegradation rate.

Compared to bare ZnO NRs, the absorption of ZnO/CuSe nanocomposites is improved in the visible region. It has been attributed to the absorption of the free carrier in the visible range^57^. The narrow bandgap of CuSe (2.38 eV) is responsible for the higher absorption of ZnO/CuSe, which confirms that the enhancement of photocatalytic performance of ZnO/CuSe is mostly owing to the accumulation of both visible light and efficient segregation of charge carriers. The narrow and wide bandgaps, respectively, of CuSe and ZnO, are responsible for the absorption of both visible and UV light. Both VB and CB of CuSe and ZnO undergo electron transport, though the VB of CuSe still retained holes. Together with the photogenerated electrons, CuSe and ZnO serve as photoelectronic reservoirs in the CB. The transfer of photo-generated holes from the valence band of ZnO/CuSe nanocomposites significantly enhances the Photocatalytic activity which directly oxidizes the organic pollutants^55^.

## Conclusions

The precipitation method successfully synthesizes the ZnO/CuSe heterojunction photocatalyst. Crystal structures analysis indicated that ZnO has a rod-like structure. The optical band gap of ZnO/CuSe ranged from 2.42 to 2.62 eV, and UV–Vis spectra confirmed that the absorption of photocatalyst has reduced. The degradation rate of MB by ZnO/CuSe photocatalyst is greater than that of ZnO. Under UV–visible irradiation, the ZnO/CuSe photocatalyst showed outstanding photocatalytic activity. The ZnO/CuSe heterojunction structure enhances the segregation of photo-induced carriers according to the photocatalytic mechanism. The reduction of photoelectrons was the primary degradation mechanism in this system.

### Supplementary Information

Supplementary Information.

## Data Availability

The datasets used and/or analyzed during the current study available from the corresponding author on reasonable request.
