# Supplementary material for: ZnO/CuSe composite-mediated bandgap modulation for enhanced photocatalytic performance against methyl blue dye
Source: Sci Rep. 2023 Nov 9;13:19580. doi: 10.1038/s41598-023-46780-y (PMC10638292; doi:10.1038/s41598-023-46780-y)
Supplement: Supplementary file 1 — Supplementary Information. [file 41598_2023_46780_MOESM1_ESM.docx]

**Supplementary Information**

**ZnO/CuSe Composite-mediated Bandgap Modulation for Enhanced Photocatalytic Performance against Methyl Blue Dye**

Khalida Mubeen^1,4^, Kashif Safeen^2^, Afshan Irshad^1,3^, Akif Safeen^5*^, Tayyaba Ghani^6^, Wiqar H Shah^7^, Rajwali Khan^8^, Khawaja Shafique Ahmad^9^, Ryan Casin^10^, Mohamed A. Rashwan^11^ and Hosam O. Elansary ^12*^, Attaullah Shah^*4^

^1^Department of Physics and Applied Mathematics, Pakistan Institute of Engineering and Applied Sciences (PIEAS), Nilore 45650, Islamabad, Pakistan

^2^ Department of Physics, Abdul Wali Khan University, Mardan, 23200, Pakistan

^3^Center for Mathematical Sciences, PIEAS, Nilore, Islamabad 45650, Pakistan

^4^National Institute of Lasers and Optoelectronics College, Pakistan Institute of Engineering and Applied Sciences, Nilore, Islamabad 45650, Pakistan

^5^ Department of Physics, University of Poonch Rawalakot, 12350, AJK, Pakistan

^6^Department of Metallurgy and Material Engineering, Pakistan Institute of Engineering and Applied Sciences (PIEAS), Nilore 45650, Islamabad, Pakistan

^7^Department of Physics, Faculty of Basic and Applied Sciences, International Islamic University H-10 Islamabad, Pakistan

^8^Department of Physics, University of Lakki Marwat, 28420, KP, Pakistan

^9^Department of Botany, University of Poonch Rawalakot, 12350, AJK, Pakistan

^10^School of Public Health, University of California, Berkeley, 2121 Berkeley Way, Berkeley, CA 94704, USA; [ryan.casini@berkeley.edu](mailto:ryan.casini@berkeley.edu) (R.C.)

^11^Department of Agricultural Engineering, College of Food and Agriculture Sciences, King Saud University, Riyadh 11451, Saudi Arabia

^12^Plant Production Department, College of Food & Agriculture Sciences, King Saud University, Riyadh 11451, Saudi Arabia; [helansary@ksu.edu.sa](mailto:helansary@ksu.edu.sa) (H.O.E.)

^*^Corresponding:

[akifsafeen@upr.edu.pk](mailto:akifsafeen@upr.edu.pk) (A.S);

[helansary@ksu.edu.sa](mailto:helansary@ksu.edu.sa) (H.O.E.)

[attashah168@gmail.com](mailto:attashah168@gmail.com) (A.S)

As a reference, the SEM images of pure CuSe, nanoparticles, ZnO nanorods and composites are shown in Fig. S1 along their elemental mapping. We conducted EDS mapping on samples of ZnO, CuSe, and ZnO/CuSe nanocomposites to provide additional insights into our findings. In Fig. S1a, the SEM image of the ZnO sample is shown in the inset. Fig. S1a1 and S1a2 display SEM images of the ZnO sample used for EDS mapping. The corresponding EDS mapping results are indicated in yellow and green, representing the presence of O and Zn in the ZnO sample, respectively. In Fig. S1 (b1 to b2), SEM images of the ZnO sample used for EDS mapping are shown. The EDS mapping results are displayed in red and purple, denoting the presence of Cu and Se in the CuSe sample, respectively. In contrast, Fig. S1c presents the SEM image used for EDS mapping of the ZnO/CuSe nanocomposite, at the same scale bar as used for ZnO (10 μm). The mapping results are indicated in different colors in Fig. S1 (c1 to c3), representing ZnO, Cu, and Se, further confirming the successful synthesis of highly pure nanomaterials. Furthermore, the inset in Fig. S1c1 illustrates the atomic ratio of the elements (Zn, O, Cu, Se) in the composite, providing solid evidence of the product's formation


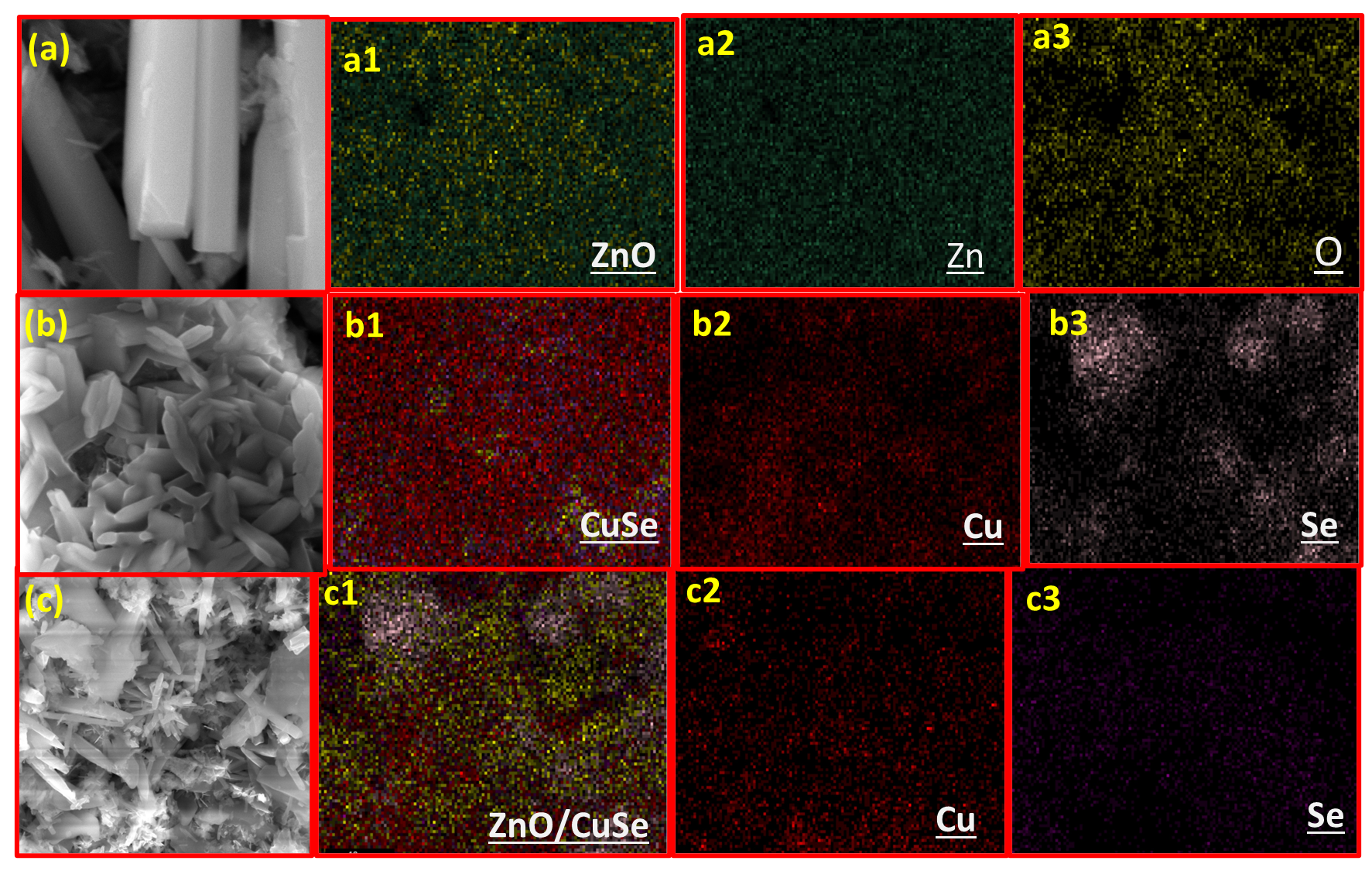


Fig. S1. (a) SEM micrograph of selected image for mapping for ZnO sample, (a1-a3) mapping results of Zn and O, (b) SEM micrograph of selected image for mapping for CuSe sample, (b1-b3) mapping results of Cu and Se (c)SEM micrograph of ZnO/CuSe nanocomposite, (c1-c3) mapping results of Cu, Se and Zn, and O elements, respectively.

The degradation of methylene blue (MB) in the absence of any catalyst primarily occurs through a series of chemical and environmental processes ^1^. One of the prominent factors leading to MB degradation is exposure to light, particularly ultraviolet (UV) light. The degradation of the MB without a catalyst is shown in Fig. S2. When MB molecules absorb photons of sufficient energy from UV or visible light, they can undergo photochemical reactions. These reactions result in the breaking of chemical bonds within the MB molecule, leading to the formation of various degradation products ^2^. This process, known as photodegradation, gradually diminishes the color and effectiveness of MB. Additionally, MB is susceptible to oxidation in the presence of atmospheric oxygen (O_2_). Oxygen can react with MB molecules, leading to oxidative degradation, which can further contribute to the loss of MB's color and stability. ^3^ While temperature, pH, and the presence of impurities can also influence MB's degradation rate, it is primarily the combined effects of light and oxygen exposure that led to the deterioration of MB in the absence of any catalyst. Proper storage in dark, cool, and dry conditions is essential to mitigate this degradation and maintain the dye's effectiveness over time ^4^.


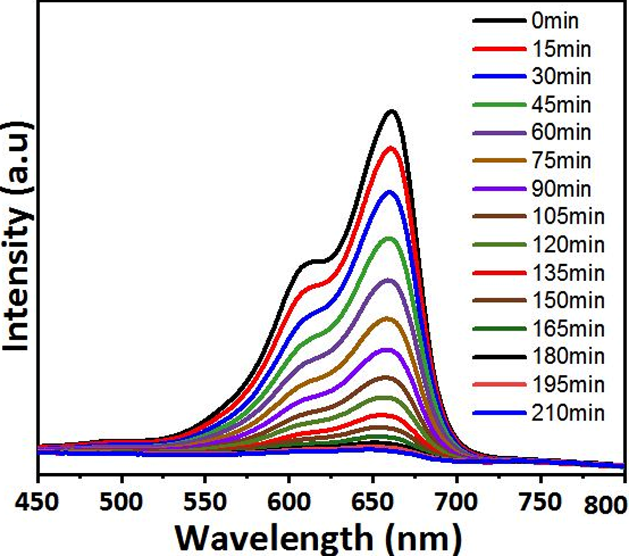


**Fig. S2:** Degradation of the MB without catalyst

Various scavengers were introduced to the photocatalytic process, namely EDTA, benzoquinone, methanol, and isopropyl alcohol, with each serving the purpose of capturing holes, superoxide radicals (•O -2), and hydroxyl radicals (•OH), respectively. The concentrations of benzoquinone and EDTA were both set at 1 mmol L^-1^, and 0.1 m/L of isopropyl alcohol was added to 400 mL of the reaction solution. The outcomes of the photocatalytic degradation of MB, in the presence of different scavengers, are illustrated in Fig. S3.


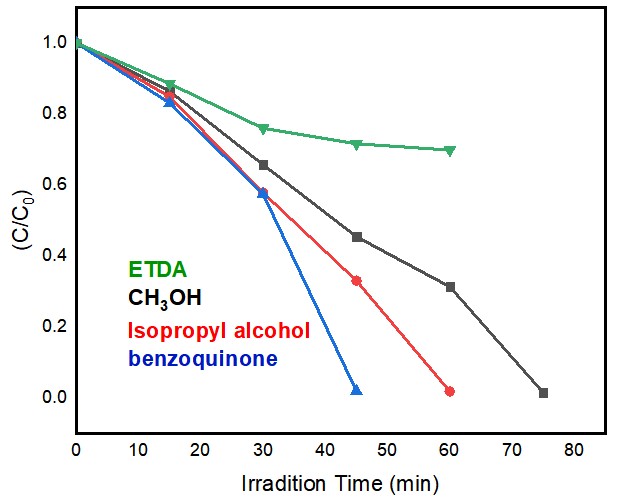


**Fig. S3:** Impact of various scavengers on the photodegradation of MB dye.

In Fig. S3, the changes in the C/C₀ ratio of MB are displayed over the irradiation period in the presence of scavengers. Specifically, when benzoquinone was introduced to the system, only 66.4% of the MB was degraded after 90 minutes, noticeably impeding the degradation compared to the 98.9% degradation observed in the absence of scavengers. The addition of isopropyl alcohol had a marginal inhibitory effect on MB degradation. Conversely, with the introduction of EDTA, the MB concentration in the system decreased rapidly, resulting in the removal of 90.3% of the MB within 90 minutes. Notably, the presence of EDTA accelerated the MB degradation process.

It was observed from the initial adsorption studies of MB dyes for 30 minutes in the absence of UV radiation that very negligible amounts of dyes were removed by the nanocomposite. Fig. S4 shows the total degradation of MB dyes with time using ZnO, CuSe, and ZnO/CuSe nanocomposite photocatalysts.


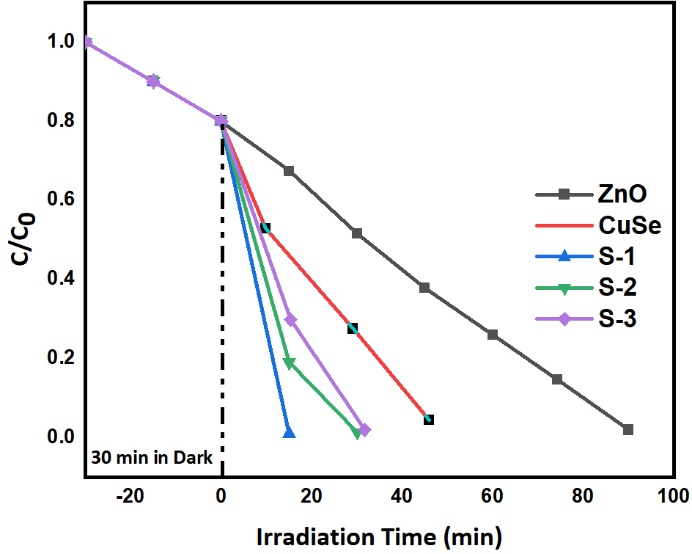


**Fig. S4**: degradation of MB with ZnO/CuSe heterostructures as a photocatalyst


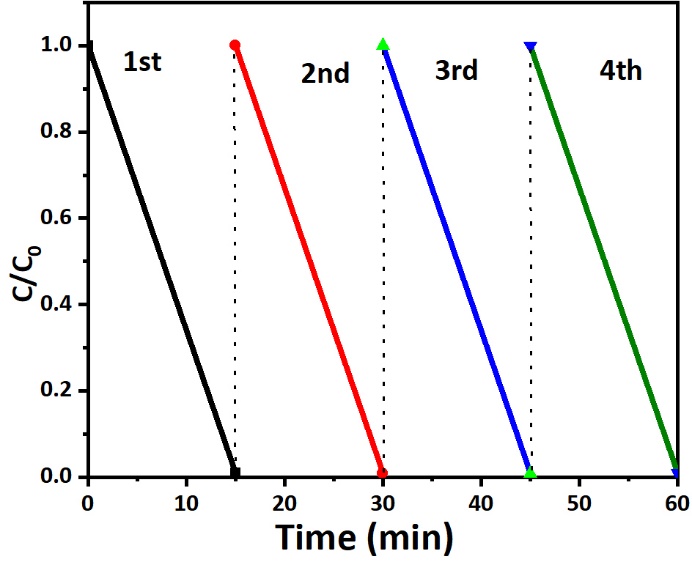


**Fig.S5:** Cyclic stability of the ZnO/CuSe nanocomposite

The cyclic stability of the ZnO/CuSe nanocomposite is shown in Fig. S5. The degradation of 98.9% of the MB in the first cycle and the lack of a substantial drop in its photocatalytic activity after three cycles show that the ZnO/CuSe nanocomposites combination demonstrated remarkable stability.

1 Khan, I. *et al.* Review on methylene blue: Its properties, uses, toxicity and photodegradation. *Water* **14**, 242 (2022).

2 Kumar, S. G. & Devi, L. G. Review on modified TiO2 photocatalysis under UV/visible light: selected results and related mechanisms on interfacial charge carrier transfer dynamics. *The Journal of physical chemistry A* **115**, 13211-13241 (2011).

3 Klosowski, E. M. *et al.* The photodynamic and direct actions of methylene blue on mitochondrial energy metabolism: A balance of the useful and harmful effects of this photosensitizer. *Free Radical Biology and Medicine* **153**, 34-53 (2020).

4 Sorbiun, M., Shayegan Mehr, E., Ramazani, A. & Taghavi Fardood, S. Biosynthesis of Ag, ZnO and bimetallic Ag/ZnO alloy nanoparticles by aqueous extract of oak fruit hull (Jaft) and investigation of photocatalytic activity of ZnO and bimetallic Ag/ZnO for degradation of basic violet 3 dye. *Journal of Materials Science: Materials in Electronics* **29**, 2806-2814 (2018).
